# Association of long-term depressive burden with incident cardiometabolic multimorbidity: a prospective study in middle-aged and older adults across three continents

**DOI:** 10.7189/jogh.16.04306

**Published:** 2026-10-02

**Authors:** Menghan Yao, Ziliang Zhang, Jiali Zhang, Meihao Wu, Dongyao Zhao, Fuyuan Wang, Yongxin Han, Yudong Miao, Guoguo Jin, Tao Li, Hao Li, Zhiping Guo

**Affiliations:** 1 Henan Key Laboratory of Chronic Disease Health Management, Fuwai Central China Cardiovascular Hospital, Henan Cardiovascular Disease Center (Central China Subcenter of National Center for Cardiovascular Diseases), Central China Fuwai Hospital of Zhengzhou University, Henan Provincial Cardiovascular Hospital, Zhengzhou, China; 2 Department of Healthy Management Center, Fuwai Central China Cardiovascular Hospital, Henan Cardiovascular Disease Center (Central China Subcenter of National Center for Cardiovascular Diseases), Central China Fuwai Hospital of Zhengzhou University, Henan Provincial Cardiovascular Hospital, Zhengzhou, China; 3 Department of Medical Laboratory, Fuwai Central China Cardiovascular Hospital, Henan Cardiovascular Disease Center (Central China Subcenter of National Center for Cardiovascular Diseases), Central China Fuwai Hospital of Zhengzhou University, Henan Provincial Cardiovascular Hospital, Zhengzhou, China; 4 College of Public Health, Zhengzhou University, Zhengzhou, China; 5 Department of Research and Foreign Affairs, Fuwai Central China Cardiovascular Hospital, Henan Cardiovascular Disease Center (Central China Subcenter of National Center for Cardiovascular Diseases), Central China Fuwai Hospital of Zhengzhou University, Henan Provincial Cardiovascular Hospital, Zhengzhou, China

**Keywords:** cardiometabolic multimorbidity, changes in depression, cumulative depressive symptoms, ageing populations

## Abstract

**Background:**

Cardiometabolic multimorbidity (CMM) is a growing concern as global population ages. Although baseline depression is a recognised risk factor for chronic diseases, the dynamic and cumulative effect of depression on CMM remains unclear. We aimed to evaluate the longitudinal association between long-term depressive burden and the risk of incident CMM.

**Methods:**

This study included 51,416 participants from three large prospective cohorts conducted in China, the USA, and Europe. Incident CMM was defined as co-occurrence of two or more of heart disease, stroke, and diabetes. Long-term depressive burden was assessed by changes in depression and cumulative depressive scores during baseline and first follow-up assessments. Cox proportional hazards model, adjusted for sociodemographic, health status, and lifestyle behaviour factors, was performed to assess incident CMM associated with long-term depressive burden. Subgroup analyses by age and sex were further conducted to evaluate potential effect modification.

**Results:**

During the follow-up period, a total of 2,689 incident CMM cases were documented: 238 in China (average follow-up = 7.61 ± 2.18 years), 829 in the USA (6.81 ± 1.89 years), and 1,622 in Europe (6.00 ± 2.20 years). Depression progression was associated with elevated risks of incident CMM in China (hazard ratio (HR) = 1.474; 95% confidence interval (CI) = 1.002–2.168), the USA (HR = 1.325; 95% CI = 1.006–1.744), and Europe (HR = 1.456; 95% CI = 1.258–1.686). Conversely, depression remission correlated with a reduced risk in China (HR = 0.558; 95% CI = 0.357–0.874) and Europe (HR = 0.764; 95% CI = 0.633–0.923). The CMM risk increased with each SD increase in cumulative depressive scores. Sex was found to modify these associations in the USA and Europe, with a more pronounced impact in females.

**Conclusions:**

Severe long-term depressive burden is associated with a higher risk of incident CMM, whereas remission from depression correlates with a relatively lower risk. These findings suggest that tracking the longitudinal dynamics of depression may serve as a valuable indicator for identifying individuals at an elevated risk of incident CMM.

Multimorbidity has emerged as a major public health challenge in the context of global population aging, with an estimated prevalence of approximately 37.2% among adults and both its prevalence and complexity increasing with advancing age [1]. Among these prevalent multimorbidity patterns, cardiometabolic multimorbidity (CMM) has emerged as one of the most consequential. Because of sharing the similar pathophysiological pathways, cardiometabolic diseases, typically including diabetes, stroke, and heart disease often co-existent [2]. CMM has been associated with a markedly higher likelihood of dementia and premature death, as well as reduced life expectancy [3–5]. Beyond these impact on survival, individuals with CMM also experience greater healthcare utilisation, including more frequent outpatient and inpatient care and a higher risk of catastrophic health expenditure [6]. Given the background of aggravating population aging and the substantial disease burden associated with CMM, the prevention and management of CMM among aging populations deserve ever more attention, not only to improve the health status and quality of life of aging individuals, but also to reduce care and financial burden on families and society. In this context, identifying modifiable and preventable risk factors for CMM have become an urgent public health priority.

CMM results from a complex interplay between physiological and environmental factors, further compounded by psychological influences [7–9]. Depression is a common and potentially modifiable mental disorder worldwide, with the prevalence of 5.7% adults experiencing depression [10]. Emerging genetic and causal evidence suggests that depression is associated with the increased risk of cardiovascular and metabolic diseases through shared immuno-inflammatory and metabolic pathways, characterised by chronic low-grade inflammation and dysregulated lipid and glucose metabolism [11–13]. Also, some unhealthy behavioural lifestyles related to depression, including prolonged sedentary time and poor sleep quality, not only influence the cardiovascular risk profiles but also interact with metabolic dysregulation to compound disease risk [14,15]. Since depression might influence incident CMM from both biological and behavioural mechanisms, intervening with depression is conducive to reducing the risk of incident CMM. According to previous studies, depression was found to elevate the risk of both cardiovascular disease and metabolic disease [16,17]. However, most of these studies relied on single-point assessments of depression, typically measured at baseline, to estimate risk related to depression, which cannot fully capture the dynamic and cumulative nature of depression and may therefore underestimate its long-term impact on the development of CMM [18]. Recent studies have found that the impact of depressive symptoms trajectories on the risk of chronic disease was more pronounced than baseline depression, underscoring the importance of repeated assessments [19–21]. Additionally, evidence remains sparse on the extent to which changes in depression influence subsequent CMM risk. The magnitude of risk reduction associated with depression remission and the excess risk associated with depression progression remains unclear, which limits the ability to evaluate the potential benefits of mental health interventions for reducing CMM risk. Moreover, an increasing number of studies have shown that the clinical importance of subthreshold depressive symptoms and their chronicity should not be disregarded, as individuals with chronic low-level symptoms may experience sustained behavioural and physiological dysregulation comparable to those meeting diagnostic thresholds [22,23]. However, most existing studies continued to rely on single-point, threshold-based assessment of depression, limiting the ability to account for the cumulative burden of depressive symptoms over time and potentially underestimating their contribution to the development of CMM.

This study aims to evaluate the longitudinal association between long-term depressive burden and the risk of incident CMM in the context of global aging. Long-term depressive burden was measured by changes in depression and cumulative depressive symptoms over time. Based on participants collected by three longitudinal cohorts, the China Health and Retirement Longitudinal Study (CHARLS), the Health and Retirement Study (HRS), and the Survey of Health, Ageing and Retirement in Europe (SHARE), this study sought to provide epidemiological evidence on the dynamic and cumulative effects of depression in the risk of incident CMM. By elucidating these associations, our findings may inform public health strategies and preventive efforts targeting depression to mitigate the growing burden of CMM and promote healthier ageing.

## METHODS

This study was conducted in strict accordance with the Guidelines for Reporting Analyses of Big Data Repositories Open to Public (GRABDROP) established by the Journal of Global Health [24]. A detailed explanation of our adherence to these guidelines is provided in the Supplementary GRABDROP Checklist in the **Online Supplementary Document**.

### Study participants

This multi-country study collected participants from three nationwide representative cohorts: CHARLS, HRS, and SHARE. To control the periodic bias, the information of participant for approximate periods was collected, say from waves 1–5 (2011, 2013, 2015, 2018, and 2020) of CHARLS, waves 10–14 (2010, 2012, 2014, 2016, and 2018) of HRS, and waves 4–8 (2012, 2014, 2016, 2018, and 2020) of SHARE. Participants with following characteristics were excluded:

• aged <45 years;

• suffering from CMM at baseline and the first follow-up;

• missing information on exposure and outcome;

• missing information on sociodemographic factors, health status, and lifestyles;

• having any emotional, nervous, or psychiatric problems at baseline.

The flowchart of participant selection was shown in **Figure 1**.

**Figure 1:**
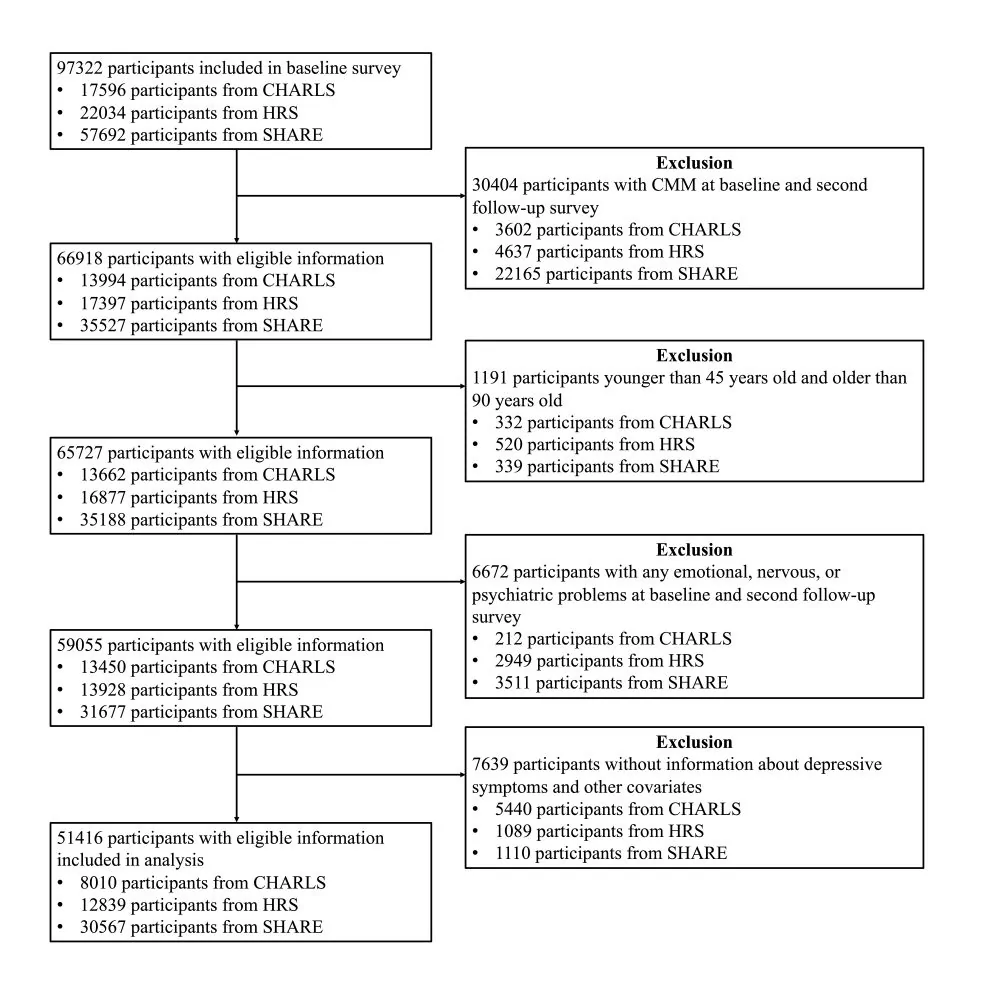
Flowchart of participants selection. CHARLS – China Health and Retirement Longitudinal Study, HRS – Health and Retirement Study, SHARE – Survey of Health, Ageing and Retirement in Europe.

### Outcome

Consistent with previous studies, cardiometabolic diseases in this study included diabetes, heart disease, and stroke [2]. These diseases were ascertained based on participants’ self-reported doctor diagnoses. Specifically, the disease definitions were:

• diabetes: diabetes or high blood glucose;

• heart disease: heart attack, coronary heart disease, angina, congestive heart failure, or other heart problems;

• stroke: stroke or transient ischemic attack (Table S1 in the **Online Supplementary Document**).

CMM was defined as the coexistence of two or more conditions. Detailed information for calculating follow-up times across cohorts are provided in Text S1 in the **Online Supplementary Document**.

### Exposure

Depression was assessed through the scores of Center for Epidemiological Studies Depression (CES-D) Scale and EURO-D Scale, utilising specific cut-offs for each country [25–27] (Table S2 in the **Online Supplementary Document**). Long-term depressive burden was assessed through two approaches, including changes in depression and cumulative depressive symptoms.

Changes in depression were categorised into four groups: stable non-depression, progression from non-depression to depression, stable depression, and remission of depression, which were assessed by the depression status of baseline and the first follow-up based on the specific cut-offs (Table S2 in the **Online Supplementary Document**).

Cumulative depressive symptoms were assessed by the depressive scores of the baseline and the first follow-up, which were calculated by the area under the curve connected by depressive scores assessed by CES-D and EURO-D [28,29]. We calculated the area under the depressive score curve (AUC) as follows:



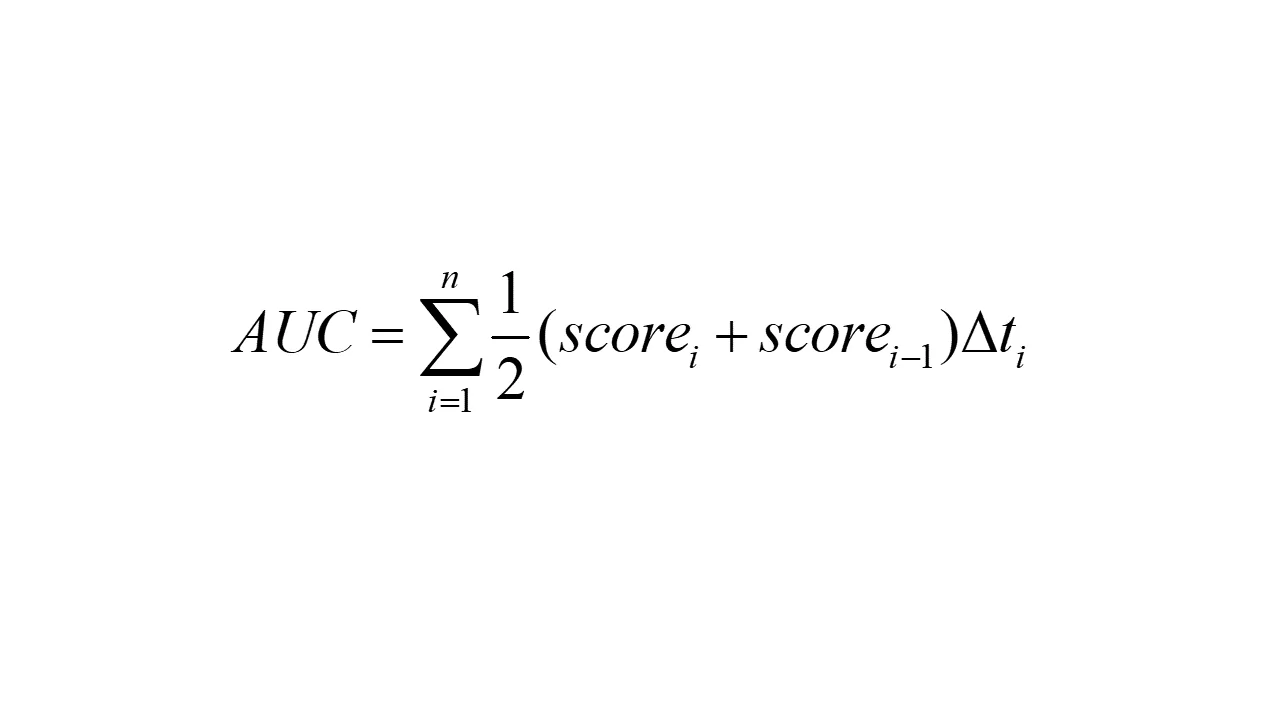



where *score_i_* denotes the CES-D score of participants at the *i*th interview and *∆t_i_* denotes the time interval between two interviews (Figure S1 in the **Online Supplementary Document**). Due to the differences in CES-D versions and study intervals, the scale of cumulative CES-D scores varies among countries. Therefore, the standardised z-score was employed in the primary analysis.

### Other covariates

Other factors that might influence the association between long-term depressive burden and incident CMM were categorised into three groups: sociodemographic factors (age, sex, marital status, and education), health status factors (history of other chronic diseases and baseline BMI category), and lifestyle factors (smoking status, alcohol consumption frequency, and physical activity) (Table S3 in the **Online Supplementary Document**). Additionally, physical activity was excluded in China as more than 57% of participants were not asked about physical activity.

### Statistical methods

Baseline characteristics of eligible participants from three cohorts were reported and differences across changes in depression were further compared through χ^2^ test for categorical variables and analysis of variance for continuous variables.

Cox proportional hazard models were used to estimate the association of long-term depressive burden with the risk of incident CMM. Specifically, long-term depressive burden was expressed through two forms, including changes in depression and cumulative depressive symptoms. The former emphasised the variations in depression status, regarding the stable non-depression and stable depression as reference groups respectively. The latter took the subthreshold depressive symptoms on incident CMM into account, which incorporated both as continuous variables, modelled per SD increment and as categorical variables based on quartiles, with the lowest quartiles as the reference category. Collinearity was evaluated using variance inflation factors (VIFs), followed by a stepwise covariate inclusion approach (Text S2 in the **Online Supplementary Document**). The proportional hazards assumption was evaluated using Schoenfeld residuals; stratified Cox models were applied when the assumption was violated. Trends across quartiles were assessed by modelling the median value of each quartile as a continuous variable. Subgroup analyses stratified by age and sex were performed to explore the potential effect modification on the association between long-term depressive burden and incident CMM, in order to identify the vulnerable populations.

Some sensitivity analyses were performed to assess the robustness of these associations from three perspective. First, to validate exposure and outcome definitions, we:

• used specific CMM patterns (heart disease, diabetes, and stroke) as outcomes;

• relied solely on baseline depressive assessments;

• evaluated cross-cohort comparability using four identical core depressive symptoms; and

• expanded the exposure definition to include the first three waves.

Second, to address potential missing data and selection biases, we:

• employed multiple imputation by chained equations (MICE) under the missing at random assumption;

• re-incorporated participants with baseline psychiatric diagnoses; and

• excluded CHARLS participants missing physical activity data.

Finally, to mitigate reverse causation and competing risks, we:

• excluded participants developing cardiometabolic diseases during the first follow-up; and

• applied the Fine-Gray model to account for the competing risk of death.

Statistical analyses were conducted using *R*, version 4.4.1 (R Foundation for Statistical Computing, Vienna, Austria), and a two-sided *P*-value <0.05 was considered statistically significant.

## RESULTS

### Baseline characteristics of participants

A total of 51,416 participants were finally included in this study, with 8,010 of CHARLS, 12,839 of HRS, and 30,567 of SHARE. During the follow-up period, a total of 2,689 incident CMM cases were documented: 238 in CHARLS with a mean follow-up of 7.61 years (standard deviation (SD) = 2.18), 829 in HRS with a mean of 6.81 years (SD = 1.89), and 1,622 in SHARE with a mean of 6.00 years (SD = 2.20). Detailed information of eligible participants was shown in **Table 1**. Additionally, participants who developed depression over the study period were more frequently female, have lower education attainment, be unmarried, and had a greater burden of chronic conditions than those who remained non-depressed. Among participants with depression at baseline, those with persistent depression were generally older, female, less educated, and had a greater burden of chronic diseases than those whose depression remitted (Tables S4–6 in the **Online Supplementary Document**).

**Table 1:** Baseline characteristics of middle-aged and older adults of CHARLS, HRS, and SHARE

Characteristic, n (%)	CHARLS	HRS	SHARE
	(n = 8,010)	(n = 12,839)	(n = 30,567)
**Age**			
<65 years old	6,118 (76.4)	6,981 (54.4)	15,882 (52.0)
≥65 years old	1,892 (23.6)	5,858 (45.6)	14,685 (48.0)
**Sex**			
Female	4,004 (50.0)	7,274 (56.7)	17,035 (55.7)
Male	4,006 (50.0)	5,565 (43.3)	13,532 (44.3)
**Education**			
Low-level	2,004 (25.0)	2,646 (20.6)	11,460 (37.5)
Medium-level	5,098 (63.6)	3,735 (29.1)	12,182 (39.9)
High-level	908 (11.4)	6,458 (50.3)	6,925 (22.6)
**Marital status**			
Other	1,192 (14.9)	4,852 (37.8)	9,249 (30.3)
Married	6,818 (85.1)	7,987 (62.2)	21,318 (69.7)
**Suffering from other disease**			
No	3,109 (38.8)	3,525 (27.5)	11,537 (37.7)
Yes	4,901 (61.2)	9,314 (72.5)	19,030 (62.3)
**BMI**			
Normal weight	4,822 (60.2)	3,719 (29.0)	11,679 (38.2)
Over weight	2,328 (29.1)	4,937 (38.5)	12,833 (42.0)
Obesity	860 (10.7)	4,183 (32.5)	6,055 (19.8)
**Smoke status**			
Never smoke	4,740 (59.2)	5,846 (45.5)	16,266 (53.2)
Previous smoker	648 (8.1)	5,138 (40.1)	8,640 (28.3)
Smoke	2,622 (32.7)	1,855 (14.4)	5,661 (18.5)
**Alcohol consumption**			
Never	5,180 (64.7)	7,388 (57.5)	11,369 (37.2)
Occasionally	651 (8.1)	3,759 (29.3)	12,258 (40.1)
Always	2,179 (27.2)	1,692 (13.2)	6,940 (22.7)
**Exercise**			
No	-	417 (3.2)	2,274 (7.4)
Yes	-	12,422 (96.8)	28,293 (92.6)

CHARLS – China Health and Retirement Longitudinal Study, BMI – body mass index, HRS – Health and Retirement Study, SHARE – Survey of Health, Ageing and Retirement in Europe

### Associations of changes in depression with incident CMM

The associations of changes in depression with incident CMM were evaluated after adjusting for sociodemographic, health status, and lifestyle behaviour factors (**Figure 2**). Compared with those who remained non-depressed in the first two surveys, non-depressed participants who progressed to depressed had higher risk of CMM, with hazard ratio (HR) of 1.474 (95% confidence interval (CI) = 1.002–2.168), 1.325 (95% CI = 1.006–1.744), and 1.456 (95% CI = 1.258–1.686) for CHARLS, HRS, and SHARE, respectively. Conversely, remission from depression to non-depression reduced the risk of incident CMM, with HR of 0.558 (95% CI = 0.357–0.874) for CHARLS and 0.764 (95% CI = 0.633–0.923). No statistically significant association was observed in HRS, with HR of 1.014 (95% CI = 0.686–1.499). Similar trends were observed throughout the stepwise covariate inclusion process (Tables S7–9 in the **Online Supplementary Document**).

**Figure 2:**
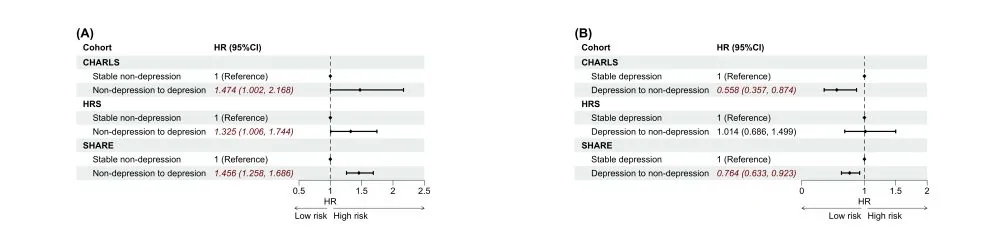
Associations of changes in depression with incident CMM across CHARLS, HRS, and SHARE. Panel A. Associations of progression from non-depression to depression with incident CMM. Panel B. Associations of remission from depression with incident CMM. HRs with *P*-value lower than 0.05 are presented in red italics. All adjusted generalised variance inflation factor values were less than 5, suggesting no substantial multicollinearity among covariates. CHARLS – China Health and Retirement Longitudinal Study, CI – confidence interval, HR – hazard ratio, HRS – Health and Retirement Study, SHARE – Survey of Health, Ageing and Retirement in Europe.

### Associations of cumulative depressive burden with incident CMM

To assess associations between cumulative depressive burden and incident CMM, cumulative depressive scores were utilised. As each SD increment of cumulative depressive scores, the risk of developing CMM increased, with HRs for 1.282 (95% CI = 1.134–1.450) in CHARLS, 1.177 (95% CI = 1.103–1.255) in HRS, and 1.231 (95% CI = 1.175–1.290) in SHARE. Compared with those in the lowest quartile, participants with cumulative depressive scores in the highest quartile had a significantly greater risk, with HRs of 1.587 (95% CI = 1.100–2.288), 1.587 (95% CI = 1.318–1.911), and 1.710 (95% CI = 1.495–1.956) in CHARLS, HRS, and SHARE, respectively (**Figure 3**). Similar trends were observed throughout the stepwise covariate inclusion process (Tables S7–9 in the **Online Supplementary Document**).

**Figure 3:**
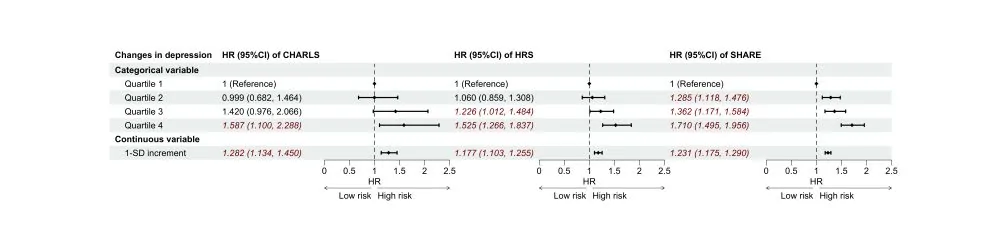
Associations between cumulative depressive symptoms and CMM incidence across CHARLS, HRS, and SHARE. HRs with *P*-value lower than 0.05 are presented in red italics. All adjusted generalised variance inflation factor values were less than 5, suggesting no substantial multicollinearity among covariates. CHARLS – China Health and Retirement Longitudinal Study, CI – confidence interval, HR – hazard ratio, HRS – Health and Retirement Study, SD – standard deviation, SHARE – Survey of Health, Ageing and Retirement in Europe.

### Subgroup analysis

In the stratified analyses by sex, progression from non-depression to depression was associated with a higher risk of incident CMM among women in CHARLS with an HR of 1.790 (95% CI = 1.064–3.010), HRS with an HR of 1.571 (95% CI = 1.127–2.189), and SHARE with an HR of 1.340 (95% CI = 1.091–1.646). The statistically significant interaction was observed in HRS, with the impact of changes in depression status on CMM incidence was more pronounced among women (**Table 2**). For depression remission, a reduced risk of CMM was observed among women in CHARLS with an HR of 0.525 (95% CI = 0.300–0.917) and SHARE with an HR of 0.609 (95% CI = 0.477–0.777), whereas no significant association was observed among men (**Table 2**).

**Table 2:** Associations between changes in depression status and CMM incidence stratified by sex*

Variable	CHARLS		HRS					SHARE			
	**HR**	**95%CI**	**HR**			**95%CI**		**HR**		**95%CI**	
**Male**											
Stable non-depression	Ref	-	Ref			-		Ref		-	
Non-depression to depression	1.142	(0.628–2.078)	0.950			(0.572–1.577)		1.574		(1.278–1.939)†	
**Female**											
Stable non-depression	Ref	-	Ref			-		Ref		-	
Non-depression to depression	1.790	(1.064–3.010)†	1.571			(1.127–2.189)†		1.340		(1.091–1.646)†	
***P* for interaction**	0.255		0.044					0.466			
**Male**											
Stable depression	Ref	-	Ref			-		Ref		-	
Depression to non-depression	0.621	(0.287–1.341)	0.630			(0.334–1.190)		1.138		(0.825–1.568)	
**Female**											
Stable depression	Ref	-	Ref			-		Ref		-	
Depression to non-depression	0.525	(0.300–0.917)†	1.248			(0.748–2.081)		0.609		(0.477–0.777)†	
***P* for interaction**	0.800			0.103					0.006		

CHARLS – China Health and Retirement Longitudinal Study, CI – confidence interval, HR – hazard ratio, HRS – Health and Retirement Study, SHARE – Survey of Health, Ageing and Retirement in Europe.

*All adjusted generalised variance inflation factor values were less than 5, suggesting no substantial multicollinearity among covariates.

†Statistically significant, *P *< 0.05.

In age-stratified analyses, associations between changes in depression status and incident CMM were broadly similar among participants aged <65 and ≥65 years. No statistically significant interactions by age were observed across cohorts, suggesting limited effect modification by age (**Table 3**). A similar pattern was observed regarding the association between cumulative depressive scores and incident CMM (Tables S10–11 in the **Online Supplementary Document**).

**Table 3:** Associations between changes in depression status and CMM incidence stratified by age*

Variable	CHARLS			HRS			SHARE		
	**HR**	**95% CI**		**HR**	**95% CI**		**HR**	**95% CI**	
**Age <65 years old**									
Stable non-depression	Ref	-		Ref	-		Ref	-	
Non-depression to depression	1.644	(1.066–2.537)†		1.138	(0.747–1.732)		1.508	(1.266–1.796)†	
**Age ≥65 years old**									
Stable non-depression	Ref	-		Ref	-		Ref	-	
Non-depression to depression	0.956	(0.398–2.294)		1.446	(1.005–2.080)†		1.356	(1.034–1.779)†	
***P* for interaction**	0.244			0.636			0.356		
**Age <65 years old**									
Stable depression	Ref	-		Ref	-		Ref	-	
Depression to non-depression	0.567	(0.334–0.963)†		0.898	(0.527–1.531)		0.911	(0.645–1.287)	
**Age ≥65 years old**									
Stable depression	Ref	-		Ref	-		Ref	-	
Depression to non-depression	0.581	(0.243–1.387)		1.113	(0.619–2.001)		0.711	(0.567–0.892)†	
***P* for interaction**	0.956			0.619			0.477		

CHARLS – China Health and Retirement Longitudinal Study, CI – confidence interval, HR – hazard ratio, HRS – Health and Retirement Study, SHARE Survey of Health, Ageing and Retirement in Europe

*All adjusted generalised variance inflation factor values were less than 5, suggesting no substantial multicollinearity among covariates.

†Statistically significant, *P *< 0.05.

### Sensitivity analysis

The associations between long-term depressive burden and incident CMM remained robust across sensitivity analyses. First, risk estimates were consistent when evaluating specific CMM patterns (Tables S12–16 in the **Online Supplementary Document**), relying on static baseline assessments, focusing on four core depressive symptoms, or expanding the exposure window (Tables S17–19 in the **Online Supplementary Document**). Second, findings were materially unaltered by multiple imputation for missing data (Table S20 in the **Online Supplementary Document**), re-incorporating participants with baseline psychiatric diagnoses, or excluding CHARLS participants missing physical activity data (Tables S21–22 in the **Online Supplementary Document**). Finally, primary trends remained stable after accounting for death as a competing risk *via* the Fine-Gray model and excluding participants who developed cardiometabolic diseases during the first follow-up (Tables S23–24 in the **Online Supplementary Document**).

## DISCUSSION

Utilising the prospective study design and longitudinal data of aging populations from China, the USA, and Europe, our study evaluated how the long-term depressive burden impacts the risk of incident CMM. We found that both progression from non-depression to depression and greater cumulative depressive burden were linked to a greater risk of CMM, which were observed across three populations. Remission from depression was associated with a reduced CMM risk. Our study provides prospective evidence that long-term depressive burden, capturing both dynamic and cumulative effect of depression, could serve as a valuable indicator for stratification and early identification of CMM risk in aging populations.

Our results showed a linkage between changes in depression and subsequent CMM across three population-based cohorts. Participants who progressed from non-depression to depression experienced a 32.5% ~ 47.4% higher risk of incident CMM compared with those who remained non-depressed, whereas remission from depression had a 23.6% ~ 44.2% lower CMM risk than individuals with persistent depression. These findings suggest that depression should be considered a dynamic, trajectory-dependent exposure rather than a single time point risk factor, whereby worsening or improvement may influence subsequent CMM risk. Several hypothetical mechanisms may provide potential theoretical frameworks to explain these associations based on previous studies. Progression from non-depression to depression may accompany by heightened HPA-axis activity and sustained cortisol elevation, promoting insulin resistance, visceral adiposity, dyslipidaemia, and hypertension, while also contributing to endothelial dysfunction and pro-atherogenic vascular changes [30,31]. Increased systemic inflammation (*e.g.* interleukin-6, C-reactive protein) caused by worsening depression status may accelerate atherosclerosis and impair glucose regulation [32,33]. Additionally, worsening symptoms often coincide with physical inactivity, poorer diet and sleep, and lower adherence to preventive and therapeutic treatment, thereby compounding the risk of developing CMM [34–37]. Conversely, remission from depression appears to attenuate these adverse biological processes, thereby mitigating CMM risk [38,39]. Due to the lack of direct biomarker measurements in our data sets, these pathways remain speculative within the scope of the current study and warrant empirical validation in future cohorts. Although previous studies have identified depression as a risk factor for CVD, diabetes, stroke, and other chronic diseases, most have relied on baseline assessments, potentially ignoring the dynamic feature of depression. By modelling changes in depression over time and investigating the association across diverse countries, our study extended the evidence that changes in depression are related to subsequent CMM. These findings suggest that recognising worsening depression is conducive to stratifying CMM risk among middle-aged and older adults, thereby highlighting the importance of incorporating routine mental health monitoring into strategies aimed at preventing CMM.

Beyond the qualitative assessment based on changes in depression, our study further quantified the association using a cumulative depressive burden metric that captures both the persistence and severity of depressive symptoms over time. Although diagnostic thresholds provide a pragmatic binary framework, growing evidence suggests that depressive symptoms below clinical cut-offs may still accumulate and exert adverse effects on overall health [23,40–42]. This metric utilises repeated assessment to better represent long-term depressive burden, mitigating the limitations of single-point measurement and threshold-based symptom definition of depression [28,29]. A dose-response association between cumulative depressive burden and subsequent CMM risk was observed across three cohorts: each standard deviation increase in cumulative depressive scores was associated with a 28.2%, 17.7%, and 23.1% higher risk of CMM in CHARLS, HRS, and SHARE, respectively. Similarly, compared with individuals in the lowest quartile, those in the highest quartile of cumulative depressive scores had markedly higher CMM risk, 58.7% in CHARLS, 58.7% in HRS, and 71.0% in SHARE. Despite substantial differences across regions in social welfare systems, cultural norms, dietary patterns, and healthcare access, the consistent direction of associations suggests that cumulative depressive burden may reflect the effect of sustained psychological distress on cardiometabolic deterioration in aging populations. The more pronounced association observed in Europe may partially relate to the higher proportion of older adults, potentially amplifying the cumulative effects of long-term psychological distress [43,44]. Taken together, our study suggests the cumulative depressive burden, calculated by the cumulative depressive scores, is a clinically interpretable metric of mental health that is associated with incident CMM, which may help identify aging populations at heightened CMM risk and inform earlier and more targeted preventive strategies.

Subgroup analyses were performed to examine the potential effect modification of age and sex on the associations between long-term depressive burden and the risk of incident CMM. In the SHARE cohort, changes in depression status had a more pronounced impact on CMM risk in females than in males. Specifically, for females, progression to depression increased the risk, with an HR of 1.340 (95% CI = 1.091–1.646), whereas remission decreased the risk, with an HR of 0.609 (95% CI = 0.477–0.777); similarly, in the HRS cohort, the effect of progression to depression on incident CMM was also more pronounced in females, with an HR of 1.571 (95% CI = 1.127–2.189). The genetic susceptibility may provide a perspective. Recent genetic epidemiological evidence utilising polygenic scores for major depression found that a higher genetic predisposition to depression conferred a greater risk of CVD incident in females, which is potentially attributed to sex-specific gene regulatory pathways that when exposed to high depressive burdens, females exhibit more pronounced proinflammatory cascades than males, further increasing the risk of CVD [45–47]. Beyond the biological mechanisms, sociocultural gender differences also amplify the cardiometabolic vulnerability in females. Disproportionate exposure to chronic psychosocial stressors, including caregiving roles and socio-economic inequalities, may exacerbate systematic inflammatory level [48]. Moreover, the sex-specific feature of sleep disturbance further compound metabolic dysregulation, which further elevated the risk of incident CMM [49]. While the biological and sociocultural mechanisms provide a plausible rationale for the sex disparities observed in our study, future precision medicine research is warranted to elucidate the exact individualised pathways underlying this vulnerability. The observed modification effects of age and sex on the association between long-term depressive burden and incident CMM should be interpreted cautiously. Stratifying the study population inevitably reduces sample sizes and statistical power within specific strata, meaning these variations could reflect random chance rather than true effect modification. Therefore, future precision medicine research with larger cohorts is warranted to elucidate the age- and sex-specific pathways underlying this vulnerability.

By utilising diverse, nationally and regionally representative cohorts across China, the USA, and Europe, our findings add epidemiological evidence that monitoring long-term depressive burden is crucial for CMM risk stratification in middle-aged and older adults. The progression to depression and cumulative depressive burden both conferred elevated risks of incident CMM, underscoring the dynamic and cumulative effect of depressive burden on incident CMM. Therefore, incorporating the longitudinal monitoring of depressive symptoms into routine chronic disease screening is essential to capture the dynamic, subclinical depressive burden that falls below formal diagnostic thresholds, thereby optimising early, stage-appropriate risk assessment. Additionally, the reduced CMM incidence observed among those whose depression remitted in China and Europe reveals an actionable window for intervention. Integrating stepped psychological and behavioural interventions, collaborative care, and other depression care into routine practice of CMM risk management may therefore enhance comprehensive health surveillance and care strategies for middle-aged and older adults [50]. Finally, when considering the broad application of these care strategies, it is important to acknowledge the inherent heterogeneity in healthcare systems and social determinants of health across populations. Although these difference warrants caution when assuming universal generalisability, the associations between long-term depressive burden and incident CMM across such diverse setting highlights the clinical value of depressive burden.

This study offers several key strengths. First, using dynamic changes in depression as an exposure provides a more nuanced understanding of how fluctuating depressive symptoms impact the incidence of CMM. Second, the inclusion of diverse populations from China, the USA, and Europe highlights the robustness of associations of incident CMM with both cumulative depressive symptoms and depression progression across populations characterised by different cultural and healthcare contexts. Additionally, the prospective cohort study design offers stronger evidence of causality, providing management from mental health perspective for CMM risk stratification. However, several limitations should be acknowledged. First, the health outcomes in our study relied on individuals’ self-reported results, which lead to an underreporting of diseases among participants, resulting in an underestimation of the true risk relationships. Our study first used questions ‘Have the hospital or doctors diagnosed it’ to some extent reduce the recall bias among middle-aged and older adults. Second, even though our study included as many as covariates that may influence the incident of CMM, there might still be some confounding variables that were not controlled. Therefore, based on the data availability, we selected data from the same or adjacent years as the baseline and end years for analysis, to minimise the periodic bias as much as possible. Third, although our prospective longitudinal design mitigates the risk of reverse causation, preclinical cardiometabolic disorders might still trigger worsening depressive symptoms prior to formal diagnosis, potentially inflating the observed associations. To address this, we conducted a sensitivity analysis excluding participants who developed incident CMM during the first two follow-up period. The results showed the robustness of our primary findings. Moreover, some factors, including genetic information, inflammatory biomarkers, *etc.*, were not included in our study due to the inaccessibility. Future precise cohorts encompassing these important factors should be established among ageing populations worldwide, in order to more accurately reflect the impact of long-term depressive burden on incident CMM and other comorbidities. Furthermore, inherent differences in scale item content, response formats, and cultural interpretations of depressive symptoms across cohorts may still influence measurement equivalence. Although our sensitivity analysis utilising four identical core symptoms demonstrated consistent associative patterns across diverse settings, future studies employing strictly cross-culturally validated diagnostic tools are warranted to further compensate for this limitation. Finally, our operationalisation of longitudinal depressive changes into a four-level categorical variable based on two assessment waves represents a simplification of potentially complex symptom trajectories, which was chosen to preserve temporal precedence and mitigate reverse causality. Future cohorts with more frequent follow-up are warranted to capture the fluctuation between two waves.

## CONCLUSIONS

In conclusion, both progression from non-depression to depression and greater cumulative depressive burden were associated with an increased risk of incident CMM, whereas remission from depression was linked to a lower risk in China and Europe. The similar patterns across populations from China, the United States, and Europe highlight the importance of incorporating depression assessment into comprehensive cardiometabolic risk assessment. These findings support integrating longitudinal monitoring of depressive symptoms into routine geriatric and primary care, with attention to the trajectory of depression. Clinically, timely recognition and effective management of depressive symptoms, including appropriate specialist care and sustained psychosocial support, may offer an additional opportunity for optimising targeted CMM screening and health surveillance in middle-aged and older adults.

## Additional material


Online Supplementary Document

## Data Availability

**Data availability:** The data sets analysed during the current study are available in GATEWAY TO GLOBAL AGING DATA, https://g2aging.org/home.
