# Supplementary material for: Association of long-term depressive burden with incident cardiometabolic multimorbidity: a prospective study in middle-aged and older adults across three continents
Source: J Glob Health. 2026 Oct 2;16:04306. doi: 10.7189/jogh.16.04306 (PMC13631972; doi:10.7189/jogh.16.04306)
Supplement: Supplementary file 1 [file jogh-16-04306-s001.pdf]

Supplement to: Yao M, Zhang Z, Zhang J, Wu M, Zhao D, Wang F, Han Y, Miao Y, Jin G, Li T, Li H, Guo Z. Association of long-term depressive burden with incident cardiometabolic multimorbidity: a prospective study in middle-aged and older adults across three continents. J Glob Health. 2026;16:04306.

Authors

Menghan Yao [1,a], Ziliang Zhang [1,a], Jiali Zhang [2], Meihao Wu [2], Dongyao Zhao [2], Fuyuan Wang [3], Yongxin Han [4], Yudong Miao [4], Guoguo Jin [1], Tao Li [5], Hao Li [1,*], Zhiping Guo [1,*]

Affiliations

[1]Henan Key Laboratory of Chronic Disease Health Management, Fuwai Central China Cardiovascular Hospital, Henan Cardiovascular Disease Center (Central China Subcenter of National Center for Cardiovascular Diseases), Central China Fuwai Hospital of Zhengzhou University, Henan Provincial Cardiovascular Hospital, Zhengzhou, China

[2]Department of Healthy Management Center, Fuwai Central China Cardiovascular Hospital, Henan Cardiovascular Disease Center (Central China Subcenter of National Center for Cardiovascular Diseases), Central China Fuwai Hospital of Zhengzhou University, Henan Provincial Cardiovascular Hospital, Zhengzhou, China

[3]Department of Medical Laboratory, Fuwai Central China Cardiovascular Hospital, Henan Cardiovascular Disease Center (Central China Subcenter of National Center for Cardiovascular Diseases), Central China Fuwai Hospital of Zhengzhou University, Henan Provincial Cardiovascular Hospital, Zhengzhou, China

[4]College of Public Health, Zhengzhou University, Zhengzhou, China

[5]Department of Research and Foreign Affairs, Fuwai Central China Cardiovascular Hospital, Henan Cardiovascular Disease Center (Central China Subcenter of National Center for Cardiovascular Diseases), Central China Fuwai Hospital of Zhengzhou University, Henan Provincial Cardiovascular Hospital, Zhengzhou, China

*** Corresponding Authors**

Zhiping Guo, Henan Key Laboratory of Chronic Disease Health Management, Fuwai Central China Cardiovascular Hospital, Central China Fuwai Hospital of Zhengzhou University, Email: gzpsohu@126.com, zpguo@zzu.edu.cn

Hao Li, Henan Key Laboratory of Chronic Disease Health Management, Fuwai Central China Cardiovascular Hospital, Central China Fuwai Hospital of Zhengzhou University, Email: leehao2004@163.com

[a] These authors contributed equally to this work.

Contents

GRABDROP Checklist. 1
STROBE Statement—Checklist of items that should be included in reports of *cohort studies*.....2
Table S1 Definitions of heart problem and stroke in CHARLS, HRS, and SHARE. 4
Table S2 Details of CES-D scale and EURO-D. 5
Table S3. Descriptive information for all covariates included in the models 6
Table S4 Baseline characteristic of participants of CHARLS, according to the changes in depression status during the baseline and the second follow-up survey. 8
Table S5 Baseline characteristic of participants of HRS, according to the changes in depression status during the baseline and the second follow-up survey. 10
Table S6 Baseline characteristic of participants of SHARE, according to the changes in depression status during the baseline and the second follow-up survey. 12
Table S7 Associations of long-term depressive burden with incident CMM with stepwise adjustment for sociodemographic, health, and lifestyle factors in CHARLS 14
Table S8 Associations of long-term depressive burden with incident CMM with stepwise adjustment for sociodemographic, health, and lifestyle factors in HRS 15
Table S9 Associations of long-term depressive burden with incident CMM with stepwise adjustment for sociodemographic, health, and lifestyle factors in SHARE 16
Table S10 Associations between changes in depression status and CMM incidence stratified by sex 17
Table S11 Associations between changes in depression status and CMM incidence stratified by age 18
Table S12 Associations between changes in depression status and incidence of specific CMM pattern. 19
Table S13 Associations between cumulative depressive symptoms and incidence of specific CMM pattern. 20
Table S14 Associations of heart disease incidence with changes in depression and cumulative depressive scores. 21
Table S15 Associations of diabetes incidence with changes in depression and cumulative depressive scores. 22
Table S16 Associations of stroke incidence with changes in depression and cumulative depressive scores. 23
Table S17 Associations between baseline depression and CMM incidence. 24
Table S18 Associations of four core depressive symptoms with the risk of incident CMM. 25
Table S19 Associations of long-term depressive burden of three waves with incident CMM. 26
Table S20 Associations of CMM incidence with changes in depression and cumulative depressive scores with complete dataset after multiple imputation. 27
Table S21 The results of primary analysis and sensitivity analysis incorporating participant who reported any emotional, nervous, or psychiatric problems by a doctor at baseline. 28
Table S22 Associations of long-term depressive burden with incident CMM in CHARLS excluding participants without physical activity information 29
Table S23 Association between long-term depressive burden and incident CMM, accounting for the competing risk of death 30
Table S24 Associations of long-term depressive burden with incident CMM by excluding

participants with any CMDs in baseline and the first follow-up..31
Figure S1. Calculation of cumulative depressive symptoms. The gray part is the cumulative burden of depressive symptoms of each individual. 32
Text S1. Calculation of follow-up time...33
Text S2. Collinearity Evaluation and Covariate Adjustment Strategy..34
Text S3. Detailed Information for Sensitivity Analyses 35

GRABDROP Checklist.

JoGH guideline items

1. Please list all papers published by each co-author in previous 3 years that were based on secondary analysis of a big data repository

Du, Q., **Yao, M.**, Wang, W., Wang, J., Li, S., Lu, K., ... & Ma, Y. (2025). Association between Multimorbidity and depression in older adults: evidence from six large longitudinal cohorts. The American Journal of Geriatric Psychiatry, 33(6), 702-715.

Li, C., Wang, W., Wei, Y., Lu, K., Wang, J., **Yao, M**., ... & Ma, Y. (2025). Association between cognitive decline and depression in middle-aged and older adults: findings from six large cohorts in different countries. Journal of Affective Disorders, 371, 215-223.

Wang, J., Wang, W., Liu, Y., **Yao, M.**, Du, Q., Wei, Y., ... & Ma, Y. (2024). Relationship between cognitive function and sleep quality in middle-aged and older adults for minimizing disparities and achieving equity in health: evidence from multiple nationwide cohorts. Archives of Gerontology and Geriatrics, 127, 105585.

2. Please explain the key elements of your study design and the use of the available datasets that make your study an original scientific contribution

Thank you for providing the opportunity to highlight the originality of our work. The scientific originality of our study lies in its innovative longitudinal design and the harmonization of multi-continental datasets. The key elements are detailed as follows:

1) Using dynamic changes in depression as an exposure provides a more nuanced understanding of how fluctuating depressive symptoms impact the incidence of CMM.

2) The inclusion of diverse populations from China, the United States, and Europe highlights the robustness of associations of incident CMM with both cumulative depressive symptoms and depression progression across populations characterized by different cultural and healthcare contexts.

3) The prospective cohort study design offers stronger evidence of causality, providing management from mental health perspective for CMM risk stratification.

3. Please list all publications that addressed similar research questions in the same dataset and indicate where you cited them in your paper

Previous research mainly investigated the impact of depression on the incidence of single cardiometabolic disease. Our study was designed to evaluate how long-term depressive burden affect the incident CMM. We have cited these relevant previous studies in Introduction and Discussion section of the manuscript.

Min, J., Cao, Z., Chen, H., Wang, X., & Xu, C. (2024). Trajectories of depressive symptoms and risk of cardiovascular disease, cancer and mortality: a prospective cohort study. General Psychiatry, 37(3), e101456.

Yan, R., Hu, Y., Yang, J., Wang, H., Wang, Y., & Song, G. (2025). Depressive symptoms trajectories and cardiovascular disease in Chinese middle-aged and older adults: A longitudinal cohort study. Journal of Affective Disorders, 380, 456-465.

Li, H., Zheng, D., Li, Z., Wu, Z., & Guo, X. (2019) Association of Depressive Symptoms With Incident Cardiovascular Diseases in Middle-Aged and Older Chinese Adults. JAMA Network Open. 2(12):e1916591.

4. Please explain how you addressed multiple testing through an appropriately rigorous statistical threshold and indicate this in the methods section

To quantify the magnitude of associations in our primary analyses, we reported effect estimates as hazard ratios (HRs) with corresponding 95% confidence intervals (CIs). And a two-sided P value <0.05 was considered statistically significant.

5. Please declare to what extent have AI chatbots been used in developing your paper and to which parts of the paper did they contribute

No AI chatbots has been used in developing this paper.

STROBE Statement—Checklist of items that should be included in reports of *cohort studies*

	Item No	Recommendation	Page No
**Title and abstract**	1	(*a*) Indicate the study's design with a commonly used term in the title or the abstract	1-2
		(*b*) Provide in the abstract an informative and balanced summary of what was done and what was found	1-2
**Introduction**			
Background/rationale	2	Explain the scientific background and rationale for the investigation being reported	3-5
Objectives	3	State specific objectives, including any prespecified hypotheses	4-5
**Methods**			
Study design	4	Present key elements of study design early in the paper	5
Setting	5	Describe the setting, locations, and relevant dates, including periods of recruitment, exposure, follow-up, and data collection	5-8
Participants	6	(*a*) Give the eligibility criteria, and the sources and methods of selection of participants. Describe methods of follow-up	5-6
		(*b*) For matched studies, give matching criteria and number of exposed and unexposed	5-8, 10
Variables	7	Clearly define all outcomes, exposures, predictors, potential confounders, and effect modifiers. Give diagnostic criteria, if applicable	6-8
Data sources/ measurement	8*	For each variable of interest, give sources of data and details of methods of assessment (measurement). Describe comparability of assessment methods if there is more than one group	7-8
Bias	9	Describe any efforts to address potential sources of bias	9
Study size	10	Explain how the study size was arrived at	5-6
Quantitative variables	11	Explain how quantitative variables were handled in the analyses. If applicable, describe which groupings were chosen and why	6-9
Statistical methods	12	(*a*) Describe all statistical methods, including those used to control for confounding	8-9
		(*b*) Describe any methods used to examine subgroups and interactions	
		(*c*) Explain how missing data were addressed	
		(*d*) If applicable, explain how loss to follow-up was addressed	
		(*e*) Describe any sensitivity analyses	
**Results**			
Participants	13*	(a) Report numbers of individuals at each stage of study—eg numbers potentially eligible, examined for eligibility, confirmed eligible, included in the study, completing follow-up, and analysed	10
		(b) Give reasons for non-participation at each stage	5-6
		(c) Consider use of a flow diagram	5-6

Descriptive data	14*	(a) Give characteristics of study participants (eg demographic, clinical, social) and information on exposures and potential confounders	10-11
		(b) Indicate number of participants with missing data for each variable of interest	5-6
		(c) Summarise follow-up time (eg, average and total amount)	10
Outcome data	15*	Report numbers of outcome events or summary measures over time	10-11

Main results	16	(*a*) Give unadjusted estimates and, if applicable, confounder-adjusted estimates and their precision (eg, 95% confidence interval). Make clear which confounders were adjusted for and why they were included	11-14
		(*b*) Report category boundaries when continuous variables were categorized	11-14
		(*c*) If relevant, consider translating estimates of relative risk into absolute risk for a meaningful time period	-
Other analyses	17	Report other analyses done—eg analyses of subgroups and interactions, and sensitivity analyses	14-15
**Discussion**			
Key results	18	Summarise key results with reference to study objectives	15-20
Limitations	19	Discuss limitations of the study, taking into account sources of potential bias or imprecision. Discuss both direction and magnitude of any potential bias	19-20
Interpretation	20	Give a cautious overall interpretation of results considering objectives, limitations, multiplicity of analyses, results from similar studies, and other relevant evidence	18-20
Generalisability	21	Discuss the generalisability (external validity) of the study results	18-20
**Other information**			
Funding	22	Give the source of funding and the role of the funders for the present study and, if applicable, for the original study on which the present article is based	21

*Give information separately for exposed and unexposed groups.

Note: An Explanation and Elaboration article discusses each checklist item and gives methodological background and published examples of transparent reporting. The STROBE checklist is best used in conjunction with this article (freely available on the Web sites of PLoS Medicine at http://www.plosmedicine.org/, Annals of Internal Medicine at http://www.annals.org/, and Epidemiology at http://www.epidem.com/). Information on the STROBE Initiative is available at http://www.strobe-statement.org.

Table S1 Definitions of heart problem and stroke in CHARLS, HRS, and SHARE.

Cohort	Heart problem	Diabetes	Stroke
CHARLS	Have you been diagnosed with heart attack, coronary heart disease, angina, congestive heart failure, or other heart problems by a doctor?	Have you been diagnosed with diabetes or high blood sugar?	Have you been diagnosed with stroke by a doctor?
HRS	Has a doctor ever told you that you have heart attack, coronary heart disease, angina, congestive heart failure, or other heart problems?	Has a doctor ever told you that you have diabetes or high blood sugar?	Has a doctor ever told you that you have stroke or transient ischemic attack (TIA)?
SHARE	Whether a doctor has told the respondent they had or currently have a heart attack, including myocardial infarction or coronary thrombosis, or any other heart problem, including congestive heart failure?	Whether a doctor has told the respondent that had or currently have diabetes or high blood sugar?	Whether a doctor has told the respondent they had or currently have a stroke or cerebral vascular disease?

Table S2 Details of CES-D scale and EURO-D.

Country and region	Cohort	Scale	Total score	Cutoff score
China	CHARLS	CES-D 10	30	10
United States	HRS	CES-D 8	8	4
Europe	SHARE	EURO-D	12	4

Table S3. Descriptive information for all covariates included in the models

Covariates	Description
**Exposure**	
Changes in depression	Stable non-depression; progression from non-depression to depression; remission from depression to non-depression; stable depression.
Cumulative depressive symptoms	The area under the curve connected by the depressive scores assessed by CES-D. $$AUC = \sum_{i=1}^{n} \frac{1}{2}(score_i + score_{i-1})\Delta t_i$$ where $score_i$ denotes the CES-D score of participants at the *i*th interview and Δt_i denotes the time interval between two interviews
**Sociodemographic factors**	
Age	Between the ages of 45 and 65 or over 65
Sex	Female or male
Marital status	Married/ Partnered or others
Education	Low (Less than lower secondary education); Medium (upper secondary education& vocational training); High (tertiary education)
**Health status factors**	
Baseline BMI category	Underweight or Normal (less than 24.9kg/m^2); Overweight (from 25 to 29.9 kg/m^2); Obesity (greater than 30 kg/m^2) in the US, Mexico, and the UK. Underweight or Normal (less than 22.9kg/m^2); Overweight (from 23 to 24.9kg/m^2); Obesity (greater than 25kg/m^2) in China and South Korea according to BMI categorization for Asians.
Chronic disease history	Whether participants suffer from chronic disease in addition to CVD, diabetes, and stroke (yes or no).
**Lifestyle factors**	
Smoking status	Non-smokers (neither smoking now nor in the past); Previous-smokers (used to smoke in the past but currently non-smoking); Current-smokers (smoking both currently and in the past).
Alcohol consumption frequency	Never-drink (0 day a week participants drink); Seldom-drink (1-4 days a week participants drink); Usually drink (more than 4 days a week participants drink)

Physical activity	Whether participants do any vigorous, moderate, or light physical activities or not (yes or no)

Table S4 Baseline characteristic of participants of CHARLS, according to the changes in depression status during the baseline and the second follow-up survey.

Characteristic	Baseline non-depression (n=5325)			Baseline depression (n=2685)		
	Stable non-depression (n=4370)	Non-depression to depression (n=955)	*P*	Stable depression (n=1393)	Depression to non-depression (n=1292)	*P*
Age			0.602			0.264
<65 years old	3400 (77.8)	735 (77.0)		1042 (74.8)	941 (72.8)	
≥65 years old	970 (22.2)	220 (23.0)		351 (25.2)	351 (27.2)	
Sex			<0.001			<0.001
Female	1900 (43.5)	526 (55.1)		892 (64.0)	686 (53.1)	
Male	2470 (56.5)	429 (44.9)		501 (36.0)	606 (46.9)	
Education			<0.001			0.001
Low-level	912 (20.9)	234 (24.5)		485 (34.8)	373 (28.9)	
Medium-level	2804 (64.2)	628 (65.8)		837 (60.1)	829 (64.2)	
High-level	654 (15.0)	93 (9.7)		71 (5.1)	90 (7.0)	
Marital Status			0.305			0.115
Other	539 (12.3)	130 (13.6)		288 (20.7)	235 (18.2)	
Married	3831 (87.7)	825 (86.4)		1105 (79.3)	1057 (81.8)	
Suffering from other disease			<0.001			<0.001
No	2018 (46.2)	349 (36.5)		332 (23.8)	410 (31.7)	
Yes	2352 (53.8)	606 (63.5)		1061 (76.2)	882 (68.3)	
BMI			0.103			0.009
Underweight	218 (5.0)	61 (6.4)		162 (11.6)	102 (7.9)	
Normal weight	2268 (51.9)	516 (54.0)		769 (55.2)	726 (56.2)	
Over weight	1376 (31.5)	271 (28.4)		344 (24.7)	337 (26.1)	
Obesity	508 (11.6)	107 (11.2)		118 (8.5)	127 (9.8)	
Smoke			0.005			<0.001
Never smoke	2444 (55.9)	581 (60.8)		946 (67.9)	769 (59.5)	
Previous smoker	414 (9.5)	65 (6.8)		73 (5.2)	96 (7.4)	
Smoke	1512 (34.6)	309 (32.4)		374 (26.8)	427 (33.0)	

Alcohol consumption			<0.001			<0.001
Never	2637 (60.3)	651 (68.2)		1034 (74.2)	858 (66.4)	
Occasionally	389 (8.9)	72 (7.5)		97 (7.0)	93 (7.2)	
Always	1344 (30.8)	232 (24.3)		262 (18.8)	341 (26.4)	

Table S5 Baseline characteristic of participants of HRS, according to the changes in depression status during the baseline and the second follow-up survey.

Characteristic	Baseline non-depression (n=11620)			Baseline depression (n=1219)		
	Stable non-depression (n=10870)	Non-depression to depression (n=750)	P	Stable depression (n=535)	Depression to non-depression (n=684)	P
Age			<0.001			0.669
<65 years old	5740 (52.8)	448 (59.7)		344 (64.3)	449 (65.6)	
≥65 years old	5130 (47.2)	302 (40.3)		191 (35.7)	235 (34.4)	
Sex			<0.001			0.87
Female	6001 (55.2)	498 (66.4)		342 (63.9)	433 (63.3)	
Male	4869 (44.8)	252 (33.6)		193 (36.1)	251 (36.7)	
Education			<0.001			0.001
Low-level	1960 (18.0)	258 (34.4)		218 (40.7)	210 (30.7)	
Medium-level	3172 (29.2)	214 (28.5)		147 (27.5)	202 (29.5)	
High-level	5738 (52.8)	278 (37.1)		170 (31.8)	272 (39.8)	
Marital Status			<0.001			0.003
Other	3818 (35.1)	372 (49.6)		317 (59.3)	345 (50.4)	
Married	7052 (64.9)	378 (50.4)		218 (40.7)	339 (49.6)	
Suffering from other disease			<0.001			0.003
No	3095 (28.5)	155 (20.7)		99 (18.5)	176 (25.7)	
Yes	7775 (71.5)	595 (79.3)		436 (81.5)	508 (74.3)	
BMI			0.062			0.266
Normal weight	3175 (29.2)	211 (28.1)		144 (26.9)	189 (27.6)	
Over weight	4254 (39.1)	271 (36.1)		170 (31.8)	242 (35.4)	
Obesity	3441 (31.7)	268 (35.7)		221 (41.3)	253 (37.0)	
Smoke			<0.001			0.001
Never smoke	5014 (46.1)	314 (41.9)		196 (36.6)	322 (47.1)	
Previous smoker	4441 (40.9)	282 (37.6)		196 (36.6)	219 (32.0)	
Smoke	1415 (13.0)	154 (20.5)		143 (26.7)	143 (20.9)	
Alcohol consumption			<0.001			0.568

Never	6123 (56.3)	485 (64.7)		350 (65.4)	430 (62.9)	
Occasionally	3216 (29.6)	202 (26.9)		146 (27.3)	195 (28.5)	
Always	1531 (14.1)	63 (8.4)		39 (7.3)	59 (8.6)	
Exercise			<0.001			<0.001
No	287 (2.6)	38 (5.1)		60 (11.2)	32 (4.7)	
Yes	10583 (97.4)	712 (94.9)		475 (88.8)	652 (95.3)	

Table S6 Baseline characteristic of participants of SHARE, according to the changes in depression status during the baseline and the second follow-up survey.

Characteristic	Baseline non-depression (n=23910)			Baseline depression (n=6657)		
	Stable depression (n=20374)	Non-depression to depression (n=3536)	P	Stable depression (n=3340)	Depression to non-depression (n=3317)	P
Age			<0.001			<0.001
<65 years old	11028 (54.1)	1642 (46.4)		1178 (53.5)	1424 (42.9)	
≥65 years old	9346 (45.9)	1894 (53.6)		1552 (46.5)	1893 (57.1)	
Sex			<0.001			<0.001
Female	10175 (49.9)	2268 (64.1)		2143 (64.2)	2449 (73.8)	
Male	10199 (50.1)	1268 (35.9)		1197 (35.8)	868 (26.2)	
Education			<0.001			<0.001
Low-level	6839 (33.6)	1563 (44.2)		1344 (40.2)	1714 (51.7)	
Medium-level	8400 (41.2)	1331 (37.6)		1348 (40.4)	1103 (33.3)	
High-level	5135 (25.2)	642 (18.2)		648 (19.4)	500 (15.0)	
Marital Status			<0.001			0.001
Other	5635 (27.7)	1126 (31.8)		1182 (35.4)	1306 (39.4)	
Married	14739 (72.3)	2410 (68.2)		2158 (64.6)	2011 (60.6)	
Suffering from other disease			<0.001			<0.001
No	8692 (42.7)	1106 (31.3)		1030 (30.8)	709 (21.4)	
Yes	11682 (57.3)	2430 (68.7)		2310 (68.2)	2608 (78.6)	
BMI			<0.001			<0.001
Normal weight	7867 (38.6)	1317 (37.2)		1275 (38.2)	1220 (36.8)	
Over weight	8763 (43.0)	1452 (41.1)		1364 (40.8)	1254 (37.8)	
Obesity	3744 (18.4)	767 (21.7)		701 (21.0)	843 (25.4)	
Smoke			<0.001			<0.001
Never smoke	10616 (52.1)	1947 (55.1)		1781 (53.3)	1922 (57.9)	
Previous smoker	6062 (29.8)	878 (24.8)		920 (27.5)	780 (23.5)	
Smoke	3696 (18.1)	711 (20.1)		639 (19.2)	614 (18.6)	
Alcohol consumption			<0.001			<0.001

Never	6625 (32.5)	1579 (44.7)		1443 (43.2)	1722 (51.9)	
Occasionally	8872 (43.5)	1186 (33.5)		1216 (36.4)	984 (29.7)	
Always	4877 (24.0)	771 (21.8)		681 (20.4)	611 (18.4)	
Exercise			<0.001			<0.001
No	957 (4.7)	395 (11.2)		355 (10.6)	567 (17.1)	
Yes	19417 (95.3)	3141 (88.8)		2985 (89.4)	2750 (82.9)	

Table S7 Associations of long-term depressive burden with incident CMM with stepwise adjustment for sociodemographic, health, and lifestyle factors in CHARLS

Long-term depressive burden		Model 1[a,*]	Model 2[b,*]	Model 3[c,*]	Model 4[d,*]
		HR (95%CI)	HR (95%CI)	HR (95%CI)	HR (95%CI)
Changes in depression	Stable non-depression	Ref	Ref	Ref	Ref
	Non-depression to depression	1.426 (0.973,2.090)	1.447 (0.985,2.126)	**1.472 (1.001,2.164)**	**1.474 (1.002,2.168)**
	Stable depression	Ref	Ref	Ref	Ref
	Depression to non-depression	**0.532 (0.343,0.827)**	**0.547 (0.351,0.853)**	**0.571 (0.366,0.893)**	**0.558 (0.357,0.874)**
Cumulative depressive scores	Q1	Ref	Ref	Ref	Ref
	Q2	1.008 (0.690,1.473)	1.014 (0.693,1.484)	1.001 (0.683,1.466)	0.999 (0.682,1.464)
	Q3	1.380 (0.956,1.993)	1.409 (0.972,2.043)	1.419 (0.976,2.064)	1.420 (0.976,2.066)
	Q4	**1.625 (1.147,2.302)**	**1.640 (1.146,2.346)**	**1.577 (1.094,2.272)**	**1.587 (1.100,2.288)**
	1-SD increment	**1.280 (1.141,1.437)**	**1.290 (1.145,1.453)**	**1.278 (1.130,1.444)**	**1.282 (1.134,1.450)**

[a] Model 1=long-term depressive burden; [b] Model 2=Model 1+sociodemographic factors; [c] Model 3=Model 2+health status factors; [d] Model 4=Model 3+lifestyle factors. [*]All adjusted generalized variance inflation factor values were less than 5, suggesting no substantial multicollinearity among covariates.

Table S8 Associations of long-term depressive burden with incident CMM with stepwise adjustment for sociodemographic, health, and lifestyle factors in HRS

Long-term depressive burden		Model 1[a,*]	Model 2[b,*]	Model 3[c,*]	Model 4[d,*]
		HR (95%CI)	HR (95%CI)	HR (95%CI)	HR (95%CI)
Changes in depression	Stable non-depression	Ref	Ref	Ref	Ref
	Non-depression to depression	**1.340 (1.023,1.757)**	**1.449 (1.101,1.906)**	**1.368 (1.039,1.801)**	**1.325 (1.006,1.744)**
	Stable depression	Ref	Ref	Ref	Ref
	Depression to non-depression	0.897 (0.615,1.308)	0.899 (0.613,1.318)	0.943 (0.643,1.384)	1.014 (0.686,1.499)
Cumulative depressive scores	Q1	Ref	Ref	Ref	Ref
	Q2	1.088 (0.882,1.341)	1.116 (0.905,1.376)	1.087 (0.881,1.340)	1.060 (0.859,1.308)
	Q3	**1.278 (1.059,1.542)**	**1.326 (1.096,1.605)**	**1.268 (1.048,1.535)**	**1.226 (1.012,1.484)**
	Q4	**1.671 (1.404,1.989)**	**1.838 (1.532,2.207)**	**1.699 (1.414,2.041)**	**1.587 (1.318,1.911)**
	1-SD increment	**1.194 (1.125,1.267)**	**1.246 (1.170,1.326)**	**1.211 (1.137,1.291)**	**1.177 (1.103,1.255)**

[a] Model 1=long-term depressive burden; [b] Model 2=Model 1+sociodemographic factors; [c] Model 3=Model 2+health status factors; [d] Model 4=Model 3+lifestyle factors. [*]All adjusted generalized variance inflation factor values were less than 5, suggesting no substantial multicollinearity among covariates.

Table S9 Associations of long-term depressive burden with incident CMM with stepwise adjustment for sociodemographic, health, and lifestyle factors in SHARE

Long-term depressive burden		Model 1[a,*]	Model 2[b,*]	Model 3[c,*]	Model 4[d,*]
		HR (95%CI)	HR (95%CI)	HR (95%CI)	HR (95%CI)
Changes in depression	Stable non-depression	Ref	Ref	Ref	Ref
	Non-depression to depression	**1.623 (1.407, 1.872)**	**1.633 (1.412, 1.887)**	**1.538 (1.330, 1.779)**	**1.456 (1.258, 1.686)**
	Stable depression	Ref	Ref	Ref	Ref
	Depression to non-depression	**0.652 (0.542, 0.784)**	**0.702 (0.583, 0.846)**	**0.740 (0.614, 0.893)**	**0.764 (0.633, 0.923)**
Cumulative depressive scores	Q1	Ref	Ref	Ref	Ref
	Q2	**1.356 (1.181, 1.557)**	**1.373 (1.196, 1.578)**	**1.314 (1.144, 1.510)**	**1.285 (1.118, 1.476)**
	Q3	**1.499 (1.292, 1.740)**	**1.536 (1.322, 1.785)**	**1.425 (1.226, 1.657)**	**1.362 (1.171, 1.584)**
	Q4	**2.126 (1.873, 2.413)**	**2.101 (1.843, 2.396)**	**1.870 (1.639, 2.135)**	**1.710 (1.495, 1.956)**
	1-SD increment	**1.351 (1.294, 1.411)**	**1.339 (1.281, 1.401)**	**1.280 (1.223, 1.340)**	**1.231 (1.175, 1.290)**

[a] Model 1=long-term depressive burden; [b] Model 2=Model 1+sociodemographic factors; [c] Model 3=Model 2+health status factors; [d] Model 4=Model 3+lifestyle factors. [*]All adjusted generalized variance inflation factor values were less than 5, suggesting no substantial multicollinearity among covariates.

Table S10 Associations between changes in depression status and CMM incidence stratified by sex

	CHARLS		HRS		SHARE	
	HR (95%CI)	*P* for interaction	HR (95%CI)	*P* for interaction	HR (95%CI)	*P* for interaction
		0.089		0.260		0.011
**Male**						
Per SD increment	1.104 (0.912, 1.337)		**1.148 (1.044, 1.261)**		**1.136 (1.065, 1.211)**	
**Female**						
Per SD increment	**1.403 (1.193, 1.651)**		**1.193 (1.093, 1.304)**		**1.302 (1.221, 1.389)**	

Abbreviations: CHARLS China Health and Retirement Longitudinal Study; CI Confidence Interval; HR Hazard Ratio; HRS Health and Retirement Study; SHARE Survey of Health, Ageing and Retirement in Europe. All adjusted generalized variance inflation factor values were less than 5, suggesting no substantial multicollinearity among covariates.

Table S11 Associations between changes in depression status and CMM incidence stratified by age

	CHARLS		HRS		SHARE	
	HR (95%CI)	*P* for interaction	HR (95%CI)	*P* for interaction	HR (95%CI)	*P* for interaction
		0.616		0.777		0.707
**Age ＜ 65 years old**						
Per SD increment	**1.280 (1.109, 1.476)**		**1.171 (1.060, 1.293)**		**1.209 (1.115, 1.310)**	
**Age ≥ 65 years old**						
Per SD increment	1.249 (0.980, 1.592)		**1.164 (1.073, 1.264)**		**1.249 (1.178, 1.324)**	

Abbreviations: CHARLS China Health and Retirement Longitudinal Study; CI Confidence Interval; HR Hazard Ratio; HRS Health and Retirement Study; SHARE Survey of Health, Ageing and Retirement in Europe. All adjusted generalized variance inflation factor values were less than 5, suggesting no substantial multicollinearity among covariates.

Table S12 Associations between changes in depression status and incidence of specific CMM pattern.

	CHARLS		HRS		SHARE	
	HR	95%CI	HR	95%CI	HR	95%CI
**Heart disease + Stroke**						
Stable non-depression	Ref	-	Ref	-	Ref	-
Non-depression to depression	1.782	(0.951, 3.342)	**1.757**	**(1.198, 2.576)**	**1.578**	**(1.294, 1.923)**
Stable depression	Ref	-	Ref	-	Ref	-
Depression to non-depression	**0.349**	**(0.154, 0.790)**	1.145	(0.665, 1.972)	**0.696**	**(0.538, 0.901)**
**Heart disease + Diabetes**						
Stable non-depression	Ref	-	Ref	-	Ref	-
Non-depression to depression	1.440	(0.860, 2.410)	1.048	(0.732, 1.499)	**1.260**	**(1.039, 1.527)**
Stable depression	Ref	-	Ref	-	Ref	-
Depression to non-depression	**0.503**	**(0.277, 0.913)**	0.991	(0.612, 1.607)	**0.713**	**(0.563, 0.902)**
**Stroke + Diabetes**						
Stable non-depression	Ref	-	Ref	-	Ref	-
Non-depression to depression	1.103	(0.531, 2.289)	1.503	(0.858, 2.634)	**1.710**	**(1.321, 2.214)**
Stable depression	Ref	-	Ref	-	Ref	-
Depression to non-depression	0.632	(0.282, 1.419)	0.735	(0.339, 1.594)	0.752	(0.543, 1.041)
**Heart disease + Stroke + Diabetes**						
Stable non-depression	Ref	-	Ref	-	Ref	-
Non-depression to depression	0.568	(0.070, 4.587)	1.092	(0.391, 3.052)	**1.587**	**(1.165, 2.163)**
Stable depression	Ref	-	Ref	-	Ref	-
Depression to non-depression	0.207	(0.018, 2.393)	1.722	(0.427, 6.948)	**0.603**	**(0.415, 0.876)**

Abbreviations: CHARLS China Health and Retirement Longitudinal Study; CI Confidence Interval; HR Hazard Ratio; HRS Health and Retirement Study; SHARE Survey of Health, Ageing and Retirement in Europe. All adjusted generalized variance inflation factor values were less than 5, suggesting no substantial multicollinearity among covariates.

Table S13 Associations between cumulative depressive symptoms and incidence of specific CMM pattern.

Cumulative depressive symptoms	CHARLS		HRS		SHARE	
	HR	95%CI	HR	95%CI	HR	95%CI
**Heart disease + Stroke**						
Quartile 1	Ref	-	Ref	-	Ref	-
Quartile 2	1.745	(0.850, 3.582)	0.970	(0.693, 1.358)	**1.351**	**(1.106, 1.651)**
Quartile 3	**2.442**	**(1.208, 4.935)**	**1.509**	**(1.141, 1.994)**	**1.605**	**(1.301, 1.979)**
Quartile 4	**2.198**	**(1.072, 4.505)**	**1.703**	**(1.284, 2.257)**	**1.968**	**(1.629, 2.377)**
P for trend	0.041		<0.001		<0.001	
Per SD increment	**1.275**	**(1.032, 1.575)**	**1.251**	**(1.138, 1.375)**	**1.295**	**(1.215, 1.380)**
**Heart disease + Diabetes**						
Quartile 1	Ref	-	Ref	-	Ref	-
Quartile 2	1.085	(0.658, 1.790)	0.998	(0.775, 1.285)	1.175	(0.983, 1.405)
Quartile 3	1.225	(0.728, 2.062)	1.066	(0.847, 1.343)	1.142	(0.937, 1.391)
Quartile 4	**1.792**	**(1.113, 2.886)**	**1.263**	**(1.006, 1.584)**	**1.587**	**(1.340, 1.880)**
P for trend	0.006		0.003			
Per SD increment	**1.293**	**(1.102, 1.518)**	**1.123**	**(1.037, 1.217)**	**1.206**	**(1.136, 1.279)**
**Stroke + Diabetes**						
Quartile 1	Ref	-	Ref	-	Ref	-
Quartile 2	0.654	(0.329, 1.303)	0.653	(0.383, 1.116)	**1.469**	**(1.125, 1.919)**
Quartile 3	1.262	(0.670, 2.344)	1.347	(0.907, 2.001)	**1.696**	**(1.281, 2.247)**
Quartile 4	1.044	(0.548, 1.987)	1.311	(0.874, 1.966)	**2.177**	**(1.692, 2.801)**
P for trend	0.610		0.038			
Per SD increment	1.177	(0.939, 1.475)	**1.183**	**(1.032, 1.355)**	**1.367**	**(1.260, 1.483)**
**Heart disease + Stroke + Diabetes**						
Quartile 1	Ref	-	Ref	-	Ref	-
Quartile 2	1.474	(0.329, 6.618)	**0.208**	**(0.063, 0.685)**	1.366	(0.983, 1.898)
Quartile 3	1.835	(0.404, 8.337)	1.168	(0.657, 2.077)	**1.639**	**(1.167, 2.301)**
Quartile 4	0.910	(0.147, 5.626)	0.855	(0.448, 1.632)	**2.381**	**(1.770, 3.204)**
P for trend	0.803		0.955		<0.001	
Per SD increment	0.942	(0.512, 1.735)	1.044	(0.821, 1.326)	**1.412**	**(1.286, 1.549)**

Abbreviations: CHARLS China Health and Retirement Longitudinal Study; CI Confidence Interval; HR Hazard Ratio; HRS Health and Retirement Study; SHARE Survey of Health, Ageing and Retirement in Europe. All adjusted generalized variance inflation factor values were less than 5, suggesting no substantial multicollinearity among covariates.

Table S14 Associations of heart disease incidence with changes in depression and cumulative depressive scores.

	CHARLS		HRS		SHARE	
	HR	95%CI	HR	95%CI	HR	95%CI
**Changes in depression**						
Stable non-depression	Ref	-	Ref	-	Ref	-
Non-depression to depression	1.142	(0.943, 1.384)	1.104	(0.856, 1.426)	**1.314**	**(1.160, 1.490)**
Stable depression	Ref	-	Ref	-	Ref	-
Depression to non-depression	**0.636**	**(0.521, 0.778)**	1.095	(0.756, 1.584)	**0.789**	**(0.668, 0.931)**
**Cumulative depressive scores**						
Quartile 1	Ref	-	Ref	-	Ref	-
Quartile 2	1.141	(0.954, 1.364)	1.064	(0.896, 1.264)	1.041	(0.906, 1.196)
Quartile 3	1.117	(0.938, 1.330)	1.073	(0.913, 1.262)	**1.215**	**(1.094, 1.349)**
Quartile 4	**1.590**	**(1.342, 1.885)**	**1.271**	**(1.081, 1.494)**	**1.424**	**(1.275, 1.591)**
P for trend	<0.001		0.003		<0.001	
Per SD increment	**1.208**	**(1.139, 1.280)**	**1.087**	**(1.024, 1.153)**	**1.165**	**(1.120, 1.213)**

Abbreviations: CHARLS China Health and Retirement Longitudinal Study; CI Confidence Interval; HR Hazard Ratio; HRS Health and Retirement Study; SHARE Survey of Health, Ageing and Retirement in Europe. All adjusted generalized variance inflation factor values were less than 5, suggesting no substantial multicollinearity among covariates.

Table S15 Associations of diabetes incidence with changes in depression and cumulative depressive scores.

	CHARLS		HRS		SHARE	
	HR	95%CI	HR	95%CI	HR	95%CI
**Changes in depression**						
Stable non-depression	Ref	-	Ref	-	Ref	-
Non-depression to depression	1.026	(0.819, 1.285)	1.238	(0.961, 1.596)	1.009	(0.851, 1.198)
Stable depression	Ref	-	Ref	-	Ref	-
Depression to non-depression	**0.775**	**(0.617, 0.972)**	0.890	(0.615, 1.287)	0.927	(0.747, 1.149)
**Cumulative depressive scores**						
Quartile 1	Ref	-	Ref	-	Ref	-
Quartile 2	1.192	(0.976, 1.455)	1.112	(0.918, 1.347)	1.048	(0.910, 1.208)
Quartile 3	1.122	(0.919, 1.371)	**1.327**	**(1.116, 1.577)**	0.935	(0.794, 1.100)
Quartile 4	**1.570**	**(1.294, 1.904)**	**1.357**	**(1.139, 1.617)**	**1.157**	**(1.002, 1.335)**
P for trend	<0.001		0.001		0.071	
Per SD increment	**1.188**	**(1.111, 1.270)**	**1.110**	**(1.046, 1.178)**	**1.055**	**(1.000, 1.113)**

Abbreviations: CHARLS China Health and Retirement Longitudinal Study; CI Confidence Interval; HR Hazard Ratio; HRS Health and Retirement Study; SHARE Survey of Health, Ageing and Retirement in Europe. All adjusted generalized variance inflation factor values were less than 5, suggesting no substantial multicollinearity among covariates.

Table S16 Associations of stroke incidence with changes in depression and cumulative depressive scores.

	CHARLS		HRS		SHARE	
	HR	95%CI	HR	95%CI	HR	95%CI
**Changes in depression**						
Stable non-depression	Ref	-	Ref	-	Ref	-
Non-depression to depression	**1.644**	**(1.286, 2.103)**	**1.722**	**(1.250, 2.372)**	**1.634**	**(1.391, 1.919)**
Stable depression	Ref	-	Ref	-	Ref	-
Depression to non-depression	**0.505**	**(0.381, 0.670)**	0.879	(0.657, 1.412)	0.901	(0.718, 1.130)
**Cumulative depressive scores**						
Quartile 1	Ref	-	Ref	-	Ref	-
Quartile 2	1.190	(0.920, 1.539)	**1.376**	**(1.060, 1.791)**	**1.264**	**(1.080, 1.478)**
Quartile 3	**1.554**	**(1.205, 2.004)**	**1.554**	**(1.220, 1.979)**	**1.365**	**(1.151, 1.620)**
Quartile 4	**1.940**	**(1.519, 2.478)**	**1.852**	**(1.456, 2.356)**	**1.641**	**(1.407, 1.620)**
P for trend	<0.001		<0.001			
Per SD increment	**1.309**	**(1.210, 1.416)**	**1.263**	**(1.166, 1.367)**	**1.196**	**(1.132, 1.264)**

Abbreviations: CHARLS China Health and Retirement Longitudinal Study; CI Confidence Interval; HR Hazard Ratio; HRS Health and Retirement Study; SHARE Survey of Health, Ageing and Retirement in Europe. All adjusted generalized variance inflation factor values were less than 5, suggesting no substantial multicollinearity among covariates.

Table S17 Associations between baseline depression and CMM incidence.

	CHARLS		HRS		SHARE	
	HR	95%CI	HR	95%CI	HR	95%CI
**Categorical variable**						
Non-depression	Ref	-	Ref	-	Ref	-
Depression	**1.336**	**(1.057, 1.689)**	**1.520**	**(1.273, 1.816)**	**1.469**	**(1.346, 1.603)**
**Continuous variable**						
Per SD increment	**1.259**	**(1.133, 1.400)**	**1.145**	**(1.083, 1.210)**	**1.205**	**(1.162, 1.250)**

Abbreviations: CHARLS China Health and Retirement Longitudinal Study; CI Confidence Interval; HR Hazard Ratio; HRS Health and Retirement Study; SHARE Survey of Health, Ageing and Retirement in Europe. All adjusted generalized variance inflation factor values were less than 5, suggesting no substantial multicollinearity among covariates.

Table S18 Associations of four core depressive symptoms with the risk of incident CMM.

Item	CHARLS	HRS	SHARE
	HR (95%CI)	HR (95%CI)	HR (95%CI)
Felt depressed			
No	Ref	Ref	Ref
Yes	0.888 (0.685,1.149)	1.392 (1.123,1.741)	1.214 (1.096,1.344)
Have trouble in sleep			
No	Ref	Ref	Ref
Yes	0.964 (0.743,1.250)	1.171 (0.989,1.386)	1,258 (1.135,1.395)
Felt everything an effort			
No	Ref	Ref	Ref
Yes	1.273 (0.981,1.653)	**1.237 (1.063,1.439)**	**1.342 (1.211,1.486)**
Enjoyed life			
No	Ref	Ref	Ref
Yes	**0.699 (0.508,0.963)**	**0.768 (0.623,0.947)**	0.025 (0.798,1.073)

Abbreviations: CHARLS China Health and Retirement Longitudinal Study; CI Confidence Interval; HR Hazard Ratio; HRS Health and Retirement Study; SHARE Survey of Health, Ageing and Retirement in Europe. All adjusted generalized variance inflation factor values were less than 5, suggesting no substantial multicollinearity among covariates.

Table S19 Associations of long-term depressive burden of three waves with incident CMM.

Long-term depressive burden		CHARLS	HRS	SHARE
		HR (95%CI)	HR (95%CI)	HR (95%CI)
Cumulative depressive scores	Q1	Ref	Ref	Ref
	Q2	0.686 (0.418,1.125)	0.958 (0.739,1.242)	**1.265 (1.034,1.547)**
	Q3	1.391 (0.913,2.120)	1.128 (0.887,1.435)	**1.364 (1.112,1.673)**
	Q4	**2.343 (1.572,3.492)**	**1.384 (1.089,1.759)**	**1.564 (1.275,1.918)**
	1-SD increment	**1.512 (1.332,1.717)**	**1.097 (1.008,1.193)**	**1.170 (1.097,1.248)**

Abbreviations: CHARLS China Health and Retirement Longitudinal Study; CI Confidence Interval; HR Hazard Ratio; HRS Health and Retirement Study; SHARE Survey of Health, Ageing and Retirement in Europe. All adjusted generalized variance inflation factor values were less than 5, suggesting no substantial multicollinearity among covariates.

Table S20 Associations of CMM incidence with changes in depression and cumulative depressive scores with complete dataset after multiple imputation.

	CHARLS		HRS		SHARE	
	HR	95%CI	HR	95%CI	HR	95%CI
**Changes in depression**						
Stable non-depression	Ref	-	Ref	-	Ref	-
Non-depression to depression	**1.691**	**(1.196, 2.391)**	**1.321**	**(1.007, 1.731)**	**1.459**	**(1.262, 1.685)**
Stable depression	Ref	-	Ref	-	Ref	-
Depression to non-depression	**0.525**	**(0.346, 0.795)**	1.053	(0.719, 1.544)	**0.788**	**(0.655, 0.948)**
**Cumulative depressive scores**						
Quartile 1	Ref	-	Ref	-	Ref	-
Quartile 2	0.924	(0.631, 1.353)	1.071	(0.870, 1.318)	**1.320**	**(1.151, 1.513)**
Quartile 3	**1.441**	**(1.029, 2.019)**	**1.238**	**(1.025, 1.496)**	**1.353**	**(1.165, 1.572)**
Quartile 4	**1.648**	**(1.174, 2.314)**	**1.586**	**(1.320, 1.906)**	**1.720**	**(1.506, 1.964)**
P for trend	<0.001		<0.001		<0.001	
Per SD increment	**1.296**	**(1.157, 1.452)**	**1.174**	**(1.101, 1.251)**	**1.227**	**(1.172, 1.286)**

Abbreviations: CHARLS China Health and Retirement Longitudinal Study; CI Confidence Interval; HR Hazard Ratio; HRS Health and Retirement Study; SHARE Survey of Health, Ageing and Retirement in Europe. All adjusted generalized variance inflation factor values were less than 5, suggesting no substantial multicollinearity among covariates.

Table S21 The results of primary analysis and sensitivity analysis incorporating participant who reported any emotional, nervous, or psychiatric problems by a doctor at baseline.

Long-term depressive burden		CHARLS		HRS		SHARE	
		HR	95%CI	HR	95%CI	HR	95%CI
Changes in depression	Stable non-depression	Ref	-	Ref	-	Ref	-
	Non-depression to depression	**1.506**	**(1.028,2.207)**	**1.394**	**(1.120,1.735)**	**1.488**	**(1.295,1.710)**
	Stable depression	Ref	-	Ref	-	Ref	-
	Depression to non-depression	**0.560**	**(0.358,0.876)**	0.866	(0.660,1.136)	**0.795**	**(0.673,0.937)**
Cumulative depressive scores	Q1	Ref	-	Ref	-	Ref	-
	Q2	1.018	(0.696,1.488)	1.092	(0.926,1.289)	**1.296**	**(1.132,1.485)**
	Q3	1.401	(0.963,2.039)	**1.359**	**(1.124,1.643)**	**1.454**	**(1.274,1.660)**
	Q4	**1.560**	**(1.006,2.845)**	**1.764**	**(1.492,2.086)**	**1.811**	**(1.5842.070)**
	1-SD increment	**1.270**	**(1.123,1.437)**	**1.248**	**(1.182,1.319)**	**1.235**	**(1.181,1.291)**

Abbreviations: CHARLS China Health and Retirement Longitudinal Study; CI Confidence Interval; HR Hazard Ratio; HRS Health and Retirement Study; SHARE Survey of Health, Ageing and Retirement in Europe. All adjusted generalized variance inflation factor values were less than 5, suggesting no substantial multicollinearity among covariates.

Table S22 Associations of long-term depressive burden with incident CMM in CHARLS excluding participants without physical activity information

Long-term depressive burden		CHARLS	HRS	SHARE
		HR (95%CI)	HR (95%CI)	HR (95%CI)
Changes in depression	Stable non-depression	Ref	Ref	Ref
	Non-depression to depression	1.332 (0.747,2.374)	**1.325 (1.006,1.744)**	**1.456 (1.258, 1.686)**
	Stable depression	Ref	Ref	Ref
	Depression to non-depression	0.455 (0.206,1.005)	1.014 (0.686,1.499)	**0.764 (0.633, 0.923)**
Cumulative depressive scores	Q1	Ref	Ref	Ref
	Q2	**1.432 (0.814,2.518)**	1.060 (0.859,1.308)	**1.285 (1.118, 1.476)**
	Q3	**1.737 (1.001,3.014)**	**1.226 (1.012,1.484)**	**1.362 (1.171, 1.584)**
	Q4	1.431 (0.799,2.562)	**1.587 (1.318,1.911)**	**1.710 (1.495, 1.956)**
	1-SD increment	**1.312 (1.092,1.577)**	**1.177 (1.103,1.255)**	**1.231 (1.175, 1.290)**

Abbreviations: CHARLS China Health and Retirement Longitudinal Study; CI Confidence Interval; HR Hazard Ratio; HRS Health and Retirement Study; SHARE Survey of Health, Ageing and Retirement in Europe. All adjusted generalized variance inflation factor values were less than 5, suggesting no substantial multicollinearity among covariates.

Table S23 Association between long-term depressive burden and incident CMM, accounting for the competing risk of death

Long-term depressive burden		CHARLS	HRS	SHARE
		HR (95%CI)	HR (95%CI)	HR (95%CI)
Changes in depression	Stable non-depression	Ref	Ref	Ref
	Non-depression to depression	**1.520 (1.035,2.231)**	1.262 (0.941,1.691)	**1.251 (1.068,1.465)**
	Stable depression	Ref	Ref	Ref
	Depression to non-depression	**0.542 (0.345,0.850)**	1.071 (0.715,1.610)	0.836 (0.686,1.019)
Cumulative depressive scores	Q1	Ref	Ref	Ref
	Q2	1.120 (0.756,1.660)	1.044 (0.833,1.312)	**1.233 (1.066,1.427)**
	Q3	**1.625 (1.105,2.392)**	**1.235 (1.010,1.510)**	**1.269 (1.083,1.486)**
	Q4	**1.684 (1.167, 2.423)**	**1.428 (1.168,1.743)**	**1.462 (1.270,1.683)**
	1-SD increment	**1.293 (1.141,1.466)**	**1.140 (1.066,1.219)**	**1.156 (1.101,1.214)**

Abbreviations: CHARLS China Health and Retirement Longitudinal Study; CI Confidence Interval; HR Hazard Ratio; HRS Health and Retirement Study; SHARE Survey of Health, Ageing and Retirement in Europe. All adjusted generalized variance inflation factor values were less than 5, suggesting no substantial multicollinearity among covariates.

Table S24 Associations of long-term depressive burden with incident CMM by excluding participants with any CMDs in baseline and the first follow-up.

Long-term depressive burden		CHARLS	HRS	SHARE
		HR (95%CI)	HR (95%CI)	HR (95%CI)
Changes in depression	Stable non-depression	Ref	Ref	Ref
	Non-depression to depression	1.109 (0.664,1.851)	1.209 (0.648,2.257)	1.257 (0.909,1.739)
	Stable depression	Ref	Ref	Ref
	Depression to non-depression	0.603 (0.345,1.054)	2.294 (0.792,6.638)	0.668 (0.428,1.042)
Cumulative depressive scores	Q1	Ref	Ref	Ref
	Q2	0.950 (0.586,1.540)	1.519 (0.984,2.347)	1.216 (0.899,1.646)
	Q3	1.355 (0.867,2.119)	1.418 (0.928,2.167)	1.239 (0.914,1.680)
	Q4	1.513 (0.974,2.351)	**1.733 (1.139,2.636)**	**1.459 (1.061,2.006)**
	1-SD increment	**1.257 (1.081,1.463)**	1.096 (0.950,1.265)	**1.200 (1.088,1.329)**

Abbreviations: CHARLS China Health and Retirement Longitudinal Study; CI Confidence Interval; HR Hazard Ratio; HRS Health and Retirement Study; SHARE Survey of Health, Ageing and Retirement in Europe. All adjusted generalized variance inflation factor values were less than 5, suggesting no substantial multicollinearity among covariates.

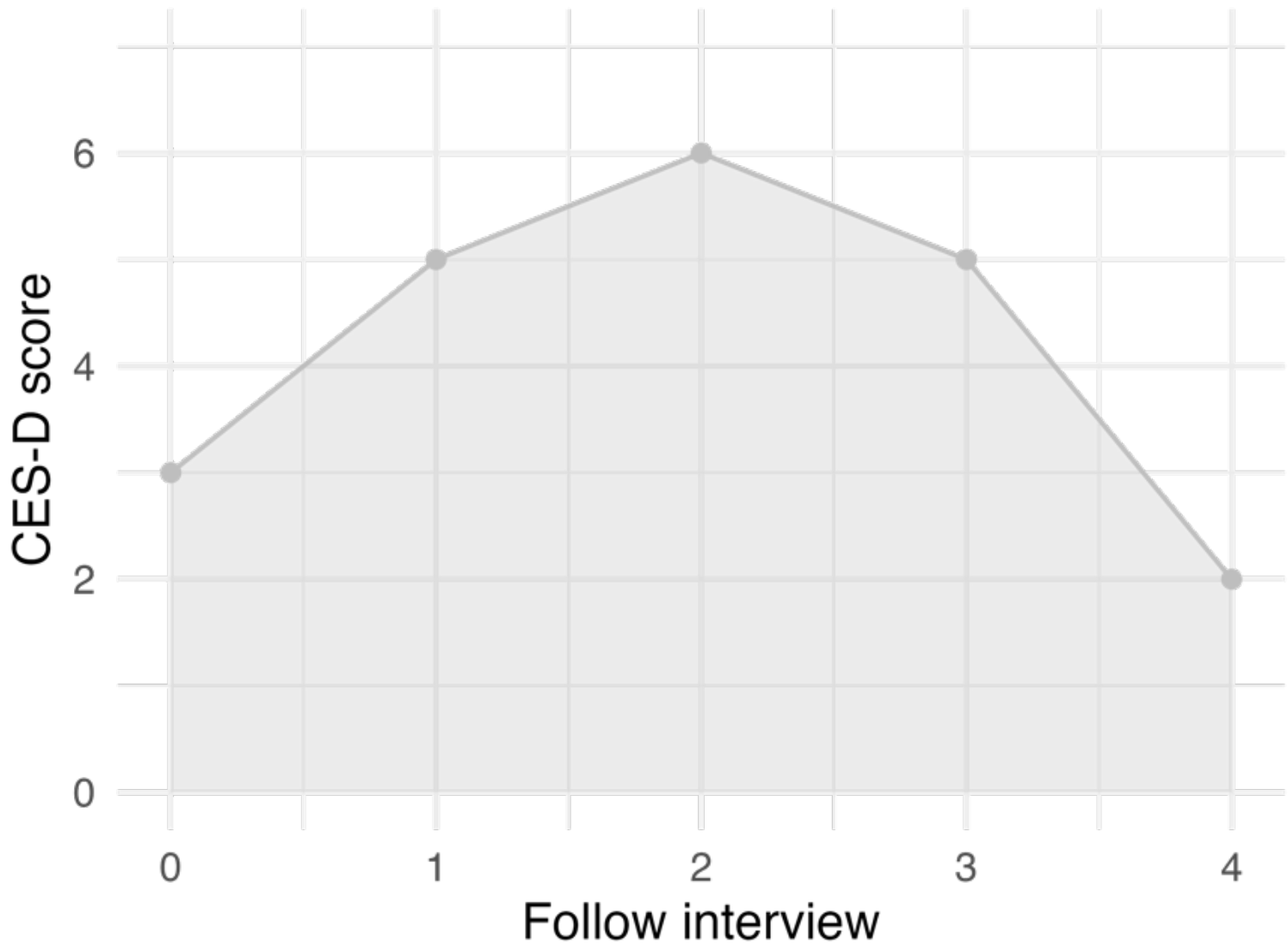

Figure S1. Calculation of cumulative depressive symptoms. The gray part is the cumulative burden of depressive symptoms of each individual.

To control the periodic bias, the information of participant for approximate periods was collected, say from waves 1-5 (2011, 2013, 2015, 2018, 2020) of CHARLS, waves 10-14 (2010, 2012, 2014, 2016, 2018) of HRS, and waves 4-8 (2012, 2014, 2016, 2018, 2020) of SHARE. To address the impact of the difference of follow-up intervals of each cohort, the depression assessment of the first two waves of each cohort were utilized to construct the cumulative depressive symptom metric as the follow-up intervals were all two years of each cohort. Before analysis, the cumulative depressive metric was standardized by these following approaches. For categorical exposure analysis, participants were classified into four quartiles (Q1-Q4) based on cohort-specific raw AUC cutoffs. Independent grouping within each cohort avoids scale-related bias, and each quartile represents an equivalent relative burden level, which ensures the comparability of stratified risks and dose-response trends. For continuous exposure analysis, we performed z-score standardization on raw AUC in each cohort, which unified data distribution and reflects individuals' relative depressive burden within each cohort.

Text S1. Calculation of follow-up time

For participants with incident CMM, the follow-up time was calculated as the interval from the baseline to the date of initial CMM onset; for all other participants, the follow-up time was calculated as the interval from the baseline to the end of the follow-up period. Considering the differences in the survey cycles and follow-up frequencies across cohorts, we calculated the average follow-up time and standard deviation separately for each cohort.

Text S2. Collinearity Evaluation and Covariate Adjustment Strategy

Before covariate inclusion, collinearity among all candidate covariates was rigorously evaluated using variance inflation factors (VIFs). Variables with evidence of significant multicollinearity (VIF > 10) were excluded from the final models, and all retained covariates had VIF values < 5, confirming the absence of severe collinearity and ensuring the stability and reliability of model parameter estimates. A stepwise covariate adjustment strategy was used as follows: first, a crude model was constructed without adjustment for any covariates (Model 1); subsequently, sociodemographic variables (Model 2), health status indicators (Model 3), and lifestyle factors (Model 4) were incorporated into the model.

Text S3. Detailed Information for Sensitivity Analyses

Some sensitivity analyses were performed to assess the robustness of these associations from three perspective. First, to test the stability of exposure and outcome definition, we: 1) evaluated the associations considering heart disease, diabetes, stroke and other specific CMM patterns as outcome; 2) assessed the associations using only baseline depressive status and scores, instead of longitudinal changes; 3) focused specifically on four identical core depressive symptoms recorded in all cohorts to test the cross-cohort comparability; and 4) expanded the exposure definition to include the first three assessment waves to capture a more comprehensive long-term depressive burden. Second, to test the impact of potential biases related to missing data and sample selection, we: 5) conducted a sensitivity analysis using multiple imputation via chained equations (MICE) under the missing at random (MAR) assumption, consistent with missing data strategies adopted in prior large-scale cohort studies; 6) re-incorporated participants who reported doctor-diagnosed psychiatric problems at baseline to evaluate potential selection bias; and 7) excluded participants in the CHARLS cohort with missing physical activity data. Finally, to test the impact of potential reverse causation and competing risk, we: 8) excluded participants who developed any cardiometabolic diseases within the first follow-up period after baseline; 9) re-analyzed the data using the Fine-Gray model to account for death as a competing risk.

The associations between long-term depressive burden and incident CMM remained robust through series sensitivity analyses. When taking heart diseases, diabetes, stroke, and other specific CMM patterns into consideration, progression from non-depression to depression would increase the risk, whereas remission from depression reduced the risk (Table S12-16 in the Online Supplementary Document). Similarly, static baseline depressive assessments, focusing on four identical core depressive symptoms across cohorts, and expanding the exposure window to the first three waves all yielded patterns consistent with our long-term dynamic assessments (Table S17-19 in the Online Supplementary Document). These findings reinforce the robust association between depressive burden and incident CMM. Third, analyses based on datasets after multiple imputation yielded consistent risk estimates with the complete-case analyses, indicating minimal bias due to missing data (Table S20 in the Online Supplementary Document). Furthermore, re-incorporating participants with baseline doctor-diagnosed psychiatric problems, or excluding those missing physical activity data in CHARLS, did not materially alter the observed associations (Table S21-22 in the Online Supplementary Document). Finally, to rigorously address methodological concerns, we accounted for death as a competing risk using the Fine-Gray model and mitigated potential reverse causation by excluding participants who developed cardiometabolic diseases within the first follow-up. In both scenarios, the overall directional trends of our primary findings remained stable (Table S23-24 in the Online Supplementary Document).
